# Large eye–head gaze shifts measured with a wearable eye tracker and an industrial camera

**DOI:** 10.3758/s13428-023-02316-w

**Published:** 2024-01-10

**Authors:** Ignace T. C. Hooge, Diederick C. Niehorster, Marcus Nyström, Roy S. Hessels

**Affiliations:** 1 Experimental Psychology, Helmholtz Institute, Utrecht University, Utrecht, The Netherlands; 2 Lund University Humanities Lab and Department of Psychology, Lund University, Lund, Sweden; 3 Lund University Humanities Lab, Lund University, Lund, Sweden

**Keywords:** Wearable eye tracking, Gaze, Head movement, Saccade

## Abstract

We built a novel setup to record large gaze shifts (up to 140^∘^). The setup consists of a wearable eye tracker and a high-speed camera with fiducial marker technology to track the head. We tested our setup by replicating findings from the classic eye–head gaze shift literature. We conclude that our new inexpensive setup is good enough to investigate the dynamics of large eye–head gaze shifts. This novel setup could be used for future research on large eye–head gaze shifts, but also for research on gaze during e.g., human interaction. We further discuss reference frames and terminology in head-free eye tracking. Despite a transition from head-fixed eye tracking to head-free gaze tracking, researchers still use head-fixed eye movement terminology when discussing world-fixed gaze phenomena. We propose to use more specific terminology for world-fixed phenomena, including gaze fixation, gaze pursuit, and gaze saccade.

## Introduction

The human retina contains a foveal area that is specialized for high-acuity color vision. The different types of eye movements serve perception of the visual world because the fovea subtends only about 1^∘^ to 2^∘^ of visual angle. Saccades enable exploration of the visual field, while other eye movements such as smooth pursuit, the opto-kinetic reflex and the vestibulo-ocular reflex serve to stabilize the retinal image during fixation. During daily behavior, such as visual search (Hooge & Erkelens, 1996; Hessels et al., 2016; Burggraaf et al., 2018; Hooge & Erkelens, 1999), driving a car (Doshi and Trivedi, 2009; Land & Tatler, 2001), assembling a tent (Sullivan et al., 2021), navigating in a crowd (Hessels et al., 2020), or preparing food or a drink in the kitchen (Land et al., 1999; Land & Hayhoe, 2001; Hayhoe et al., 2003; Macdonald & Tatler, 2018), humans fixate parts of the world that are important for the task at hand. To explore the world, they may turn the eyes relative to the head, the head relative to the body, or even the entire body with respect to the world (Radau et al., 1994; Land, 2004). Recording of combined eye–head movements is not straightforward. The present study is about the measurement of large (up to 140^∘^) horizontal eye–head gaze shifts.

Why do people make combined eye–head movements toward visual targets within their visual field? The width of the horizontal binocular field of view is approximately 220^∘^ (Harrington, 1981)^1^. The oculomotor range is about 40^∘^ to the left and the right, allowing horizontal saccades with amplitudes up to 80^∘^ (Collewijn et al., 1988). This is much smaller than the binocular field of view (220^∘^). Humans make combined eye–head movements to be able to reach the edges of their visual field in one movement. However, combined eye–head movements are also found to occur when humans make smaller gaze movements (Bartz, 1966). A consequence of such a combined eye–head movement is that post-saccadic eye-in-head eccentricity is reduced (Radau et al., 1994; Stahl, 1999). This has at least two advantages: (1) reduced post-saccadic eye eccentricity allows for exploration in all directions by means of smaller saccades and (2) to avoid long-lasting eccentric viewing, which is uncomfortable. After larger eye–head gaze shifts the eye-in-head orientation is eccentric and VOR- compensated head movement toward the target follows to center the eyes in the orbit.

Large gaze movements have been studied since the 1960s (Bartz, 1966). The main focus of the majority of the studies conducted before 2010 is on the control of the combined eye and head movements. Empirical and theoretic work revealed much of the control mechanisms that underlay the observed behavior (for a review, see Freedman 2008). Recent studies also focus on other aspects such as gaze behavior during natural tasks (Kothari et al., 2020), in VR (Bischof et al., 2023), or during interaction (Spakov et al., 2019).

## Estimating gaze

In the 1960s, eye tracking was still in its infancy, and measuring large eye–head gaze shifts was technically complicated but not impossible. Over the years, various eye-tracking techniques have been developed and the research on gaze has been carried out with all kinds of different methods.

### Techniques to assess eye orientation

Most older studies used EOG to determine eye orientation relative to the head (e.g., Bartz , 1966; Sugie and Wakakuwa , 1970; Gresty , 1973; Morasso et al. , 1973; Kasai and Zee , 1978; Zangemeister and Stark , 1982; Guitton and Volle , 1987; Pelisson et al. , 1987; Fuller 1992). EOG is non-invasive and inexpensive to conduct. This method also has many disadvantages. According to Hutton (2019): “The main disadvantage of EOG is spatial accuracy because EOG is very prone to drift artefacts over time, typically due to impedance changes at one or more of the electrodes".

After 1990, scleral coils (Collewijn et al. , 1975) have been used to estimate eye orientation relative to the world (e.g., Radau et al. , 1994; Tweed et al. , 1995; Goossens and van Opstal , 1997; Stahl , 1999; Sağlam et al. 2011). This method has the disadvantage that it is invasive, but precision (0.017^∘^, Collewijn , 2001; Malpeli 1998) is excellent even by today’s standards. Compared to EOG, the coil signal is also more accurate and does not drift.

Around the year 2000, wearable video-based eye trackers were introduced to estimate eye orientation relative to the head during head movements (e.g., Land , 2004; Boulanger et al. , 2012; Fang et al. , 2015; Sidenmark and Gellersen , 2019; Kishita et al. , 2020; Bischof et al. 2023), and the wearable eye-tracking technique is still undergoing development. In older studies, the measurement frequency was 60 Hz or less (e.g., Land , 2004; Fang et al. 2015). Wearable eye trackers operating at a frequency of 30–60 Hz are not fast enough to properly investigate saccade dynamics. The estimated bandwidth of saccades is about 75 Hz (Bahill et al., 1981, 1982), therefore, the measurement frequency should at least be twice that frequency. However, recently the frame rate of wearable eye trackers has increased (100–200 Hz). The latest development is a 500-Hz wearable eye tracker based on a micro-electro mechanical system (MEMS, Aziz et al. , 2022; Sarkar 2020).

### Techniques to assess head orientation and/or position

Most older studies used a helmet linked to a potentiometer to estimate head orientation around one axis (e.g., Bartz , 1966; Gresty , 1973; Morasso et al. , 1973; Kasai and Zee , 1978; Zangemeister and Stark , 1982; Guitton and Volle , 1987; Pelisson et al. , 1987; Fuller 1992). In the 1990s, cyclorotational coils (Collewijn et al., 1985), attached to the head have been used to determine the three-dimensional orientation of the head with respect to the world (e.g., Collewijn et al. , 1992; Epelboim et al. , 1995; Epelboim et al. , 1997; Epelboim et al. , 2019; Kowler et al. , 1992; Radau et al. , 1994; Tweed et al. , 1995; Goossens and van Opstal , 1997 ; Stahl , 1999; Sağlam et al. , 2011; Boulanger et al. 2012). To estimate three-dimensional position of the head, a group of researchers added an ingenious acoustic method (four microphones combined with a 60-kHz sound source attached to the head) to their setups (Collewijn et al. , 1992; Kowler et al. , 1992; Epelboim et al. , 1995; Epelboim et al. , 1997; Epelboim et al. , 2019). Since 2000, different methods have been used to determine head orientation and head position: A remote camera and the scene camera of the wearable eye tracker (Land, 2004), magnetic head trackers (Barabas et al., 2004; Fang et al., 2015), IR markers (Kishita et al., 2020) and VR goggles with inertial trackers calibrated via two infrared IR base stations (Sidenmark and Gellersen , 2019; Bischof et al. , 2023; Niehorster et al. , 2017), an inertial measurement unit (IMU) to estimate head orientation (Kothari et al., 2020); two IMU’s, one on the hat visor and the other as reference attached to the belt (Tomasi et al., 2016). IMU technology is still undergoing rapid development (Nazarahari & Rouhani, 2021).

### The present study

It is important to note that the different setups produce different head- and eye-orientation signals. The EOG and potentiometer setup produces eye orientation relative to the head and head orientation relative to the world. The gaze signal is then calculated from these two signals. The same applies to the signals produced by a setup consisting of a wearable eye tracker in combination with a head tracker. In a coil setup, the eye orientation relative to the head can be calculated from the gaze and the head signals.

Suppose a researcher wants to conduct research into combined large eye–head gaze shifts in 2023. Not all techniques from the past can still be applied easily. EOG is not considered good enough according to modern eye-tracking standards because it suffers from drift and inaccuracy (Hutton, 2019). The scleral coil method is very precise but is invasive, and it also requires the cornea to be anesthetized. Nowadays, the scleral coil could only pass ethical approval in very exceptional cases. Except for research conducted in academic hospitals, we are not aware of recent research using scleral coils for estimating gaze. Some modern setups are also not good enough. If they contain a low-frequency wearable eye tracker, saccade dynamics and saccade start and endpoint cannot be captured adequately. In this study, we aim to present a cheap and effective setup to record combined eye–head movements. The setup consists of a wearable eye tracker with a high frame rate (200 Hz) and an affordable industrial high-speed camera in combination with ArUco fiducial marker technology (Garrido-Jurado et al., 2014). Although the ArUco technique can determine three-dimensional orientation and position with a single marker, we have chosen a relatively simple setup to investigate large eye–head gaze shifts: rotation of the head around a vertical axis and rotation of the eyes around a vertical axis. We decided to replicate some findings from the classic combined eye–head movements literature. The main reason is that we are more interested in the dynamics of the problem than in its dimensionality. We view the latter as more of the same problem and mathematically more challenging. The dynamics of combined eye–head movements in one dimension are also of interest in applied research, such as when looking left and right while crossing the street, checking the side mirrors while driving, and during multi-party conversations.

To show that the new setup is good enough, we will replicate a number of findings from the large eye–head gaze shift literature. Moreover, we extend measurement of the gaze angle to a range from -70^∘^ to 70^∘^, which is larger than the range of most previous studies. We will discuss possibilities and limitations of our setup in comparison with other modern methods such as inertial measurement units (IMUs). Last, this article may serve as a hub to the old large eye–head-gaze shift literature for researchers new in the field of large eye–head gaze shifts. One may think of researchers using eye-tracking glasses outside the laboratory, and users of VR and AR.

**Fig. 1:**
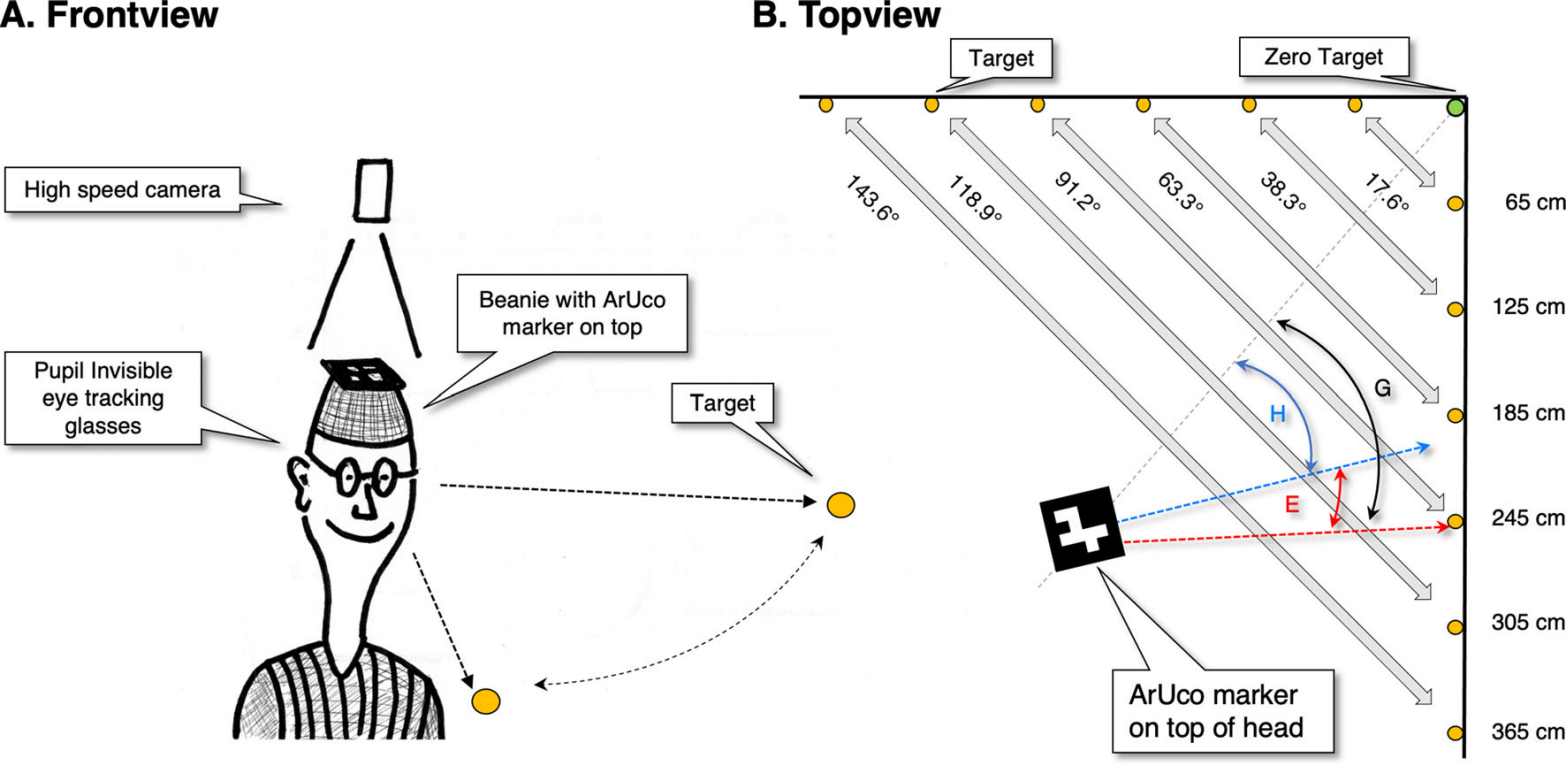
**The setup**. Panel A. The setup consists of a high speed camera (Basler ace acA2500-60um) with a 16-mm lens (1623ML12M), a beanie with an ArUco fiducial marker on top (Garrido-Jurado et al. , 2014) and the Pupil Invisible eye tracking glasses. The participant is instructed to alternately look between the left and right target (orange dot) and is allowed to rotate the head. Panel B. The distance between the participant’s head and the corner of the room is 3.43 m. The distance between pairs of targets ranges from 17.6^∘^ to 143.6^∘^. The orientation of the head (blue arc, angle denoted by H) with respect to the room is estimated by the use of the ArUco marker. The eye tracking glasses provide the eye orientation relative to the head (red arc, angled denoted by E), and the gaze angle (black arc, angle denoted by G) is computed by adding up E and H

## Methods

### Participants

Five participants (ranging in age from 25 to 57 years) took part in the experiment. They are staff members from Utrecht University and Lund University. Four of the participants are authors of the current article. Written informed consent was provided by the participants, and the experiment was conducted in accordance with the Declaration of Helsinki. This research project does not belong to the regimen of the Dutch Act on Medical Research Involving Human Subjects, and therefore there is no need for approval of a Medical Ethics Committee. However, the present study is approved by the Ethics Committee of the Faculty of Social and Behavioural Sciences of Utrecht University and filed under number 23-0081.

### The setup

The setup (Fig. 1) consists of (1) a wearable eye tracker to record eye orientation relative to the head, (2) a ceiling camera to record head orientations relative to the room by means of an ArUco marker (Garrido-Jurado et al., 2014) attached to a beanie worn by the participant and (3) 12 visual targets attached to the wall to enable the participant to make large horizontal gaze movements (up to 140^∘^). Our system provides six degrees-of-freedom measurements of head pose (orientation and position). In the present experiment, we only use one degree of rotational freedom of the head, which was sufficient to replace and extend previous research on large combined eye–head gaze shifts.

We have chosen to conduct this experiment in a reasonably large space. This has the advantage that (1) on this spatial scale, the distance between the rotation axes of the eyes and the rotation axis of the head is negligible, and (2) translations of the head have almost no impact on head and eye orientation angles. If the head translates 5 cm, this results in an absolute gaze angle error of 1.2^∘^ when fixating the closest target (at approximately 2.5 m).

The specifications of the Pupil Invisible wearable eye tracker are: a recording frequency of 220 Hz; two eye cameras; a scene camera with a resolution of 1088 pixels x 1080 pixels; a field of view of the scene camera of 82^∘^ x 82^∘^. We used the Invisible Companion application (v1.4.14-prod) to conduct the recordings.

The ceiling camera is a Basler ace acA2500-60um with a 16-mm lens (AZURE-1623ML12M). The camera filmed at 250 Hz; Image dimensions were 1152 pixels by 986 pixels; The exposure time was fixed to 3.83 ms. Video was captured at 8-bit resolution with custom software (Nyström et al., 2023; Hooge et al., 2021) that streamed the recorded frames encoded into an mp4 file using libavcodec (ffmpeg) version 5.0 and the libx264 h.264 encoder (preset: veryfast, crf: 10, pixel format: gray). We recorded at 250 Hz because this frequency is sufficiently high to capture the dynamics of head orientation. However, our system could easily be used with recording frequencies up to 1000 Hz or more, depending on the specific lens used and requirements on the noise level in the orientation data. Far higher recording frequencies using the same fiducial marker technique can be achieved using cameras that are not limited by the bandwidth of USB3, but instead transfer the image data via direct PCIe connections or high-bandwidth framegrabbers.

The targets are small circular orange stickers with a diameter of 0.8 cm, extending 0.13^∘^ to 0.18^∘^ on the retina (depending on the distance between the target and the participant). The vertical position of the targets was 97.7 cm above the floor of the room (about eye height). Because the participants varied in height, the relative height of the targets with respect to eye height ranged from 0 to 7 cm (0^∘^-1.6^∘^ for the closest target at approximately 2.45 m).

### Procedure and task

The experiment commenced by providing instructions to the participant. Besides the instruction to look at the targets, we asked the participants to try not to blink during gaze movements. Instead, we asked them to blink during periods when they were fixating the target with their head and eyes. This was followed by equipping the participant with the eye tracker. To secure the eye tracker to the participant’s head, a head strap, similar to those used in sports, was employed. A snug fit without causing any discomfort or damage was ensured. The participants were carefully placed under the ceiling camera to ensure that the ArUco marker placed on the participant’s head was visible during the largest head rotations.

We started with the calibration procedure. The participants were asked to fixate the center of the green target (Fig. 1) located in the corner of the experimental room (green rectangle [22 cm x 20 cm, 3.65^∘^ x 3.31^∘^] with a small orange circular fixation target [0.8 cm, 0.13^∘^]). This fixation provided us with the zero for the head orientation. Then the participants were asked to fixate the targets from right to left and back (in total 23 fixations). Then the actual experiment started. The participants looked from one visual target on their left side to the corresponding target on their right side. They had to repeat this between 40 and 50 times in a self-paced manner. The experiment consisted of six conditions in which the distances between the targets were varied (17.6^∘^, 38.3^∘^, 63.3^∘^, 91.2^∘^, 118.9^∘^ and 143.6^∘^). Before, after, and between conditions, the participants were asked to make five small horizontal head oscillations (similar to shaking the head side to side) while fixating the green zero target. These head oscillations served two purposes: (1) producing a signal for synchronization of the eye-tracking signal and head orientation signal from the ceiling camera, and (2) segmenting by hand the eye-tracking and head orientation signals for further processing and analysis. For a description and explanation for the synchronization procedure, see Matthis et al. (2018) and Hooge et al. (2022).

### Gaze estimation

#### Head orientation estimation

In our setup, we estimated head orientation using the ArUco marker technique. The ArUco marker attached to the participant’s head was detected with custom Python code using the *detectMarkers()* function in OpenCV 4.7.0.72, after which its pose was recovered using the perspective-n-point algorithm implementation provided by OpenCV’s *solvePnP()* function, which was run with default settings. This procedure provided us with the orientation of the ArUco marker relative to the optical axis of the camera as a rotation vector. We used the MATLAB 2022a functions *rotationVectorToMatrix.m* and *rotm2eul.m* to convert the rotation vector components to Euler angles.

#### Eye orientation estimation

We utilized the Pupil Invisible eye-tracking glasses to capture eye orientation relative to the head. To convert the eye orientation signals from pixel coordinates in the eye-tracker scene camera image to gaze directions, we followed a similar approach as outlined in (figure 4, steps 5-7 Hooge et al. 2022). The specific MATLAB code can be downloaded here https://github.com/dcnieho/HoogeetalEyeHeadGazeShifts.

#### Gaze orientation estimation

Gaze orientation (G) is calculated by combining the head orientation (H) and the eye-in-head orientation (E). Prior to combining these signals, they were resampled to a frequency of 200 Hz using *resample.m* in MATLAB r2022a. We then fit the sum of E, H, and a constant term (c) to the target positions, which represents the desired gaze orientation (G). The constant term (c) is introduced to set the zero in the corner of the room.

**Fig. 2:**
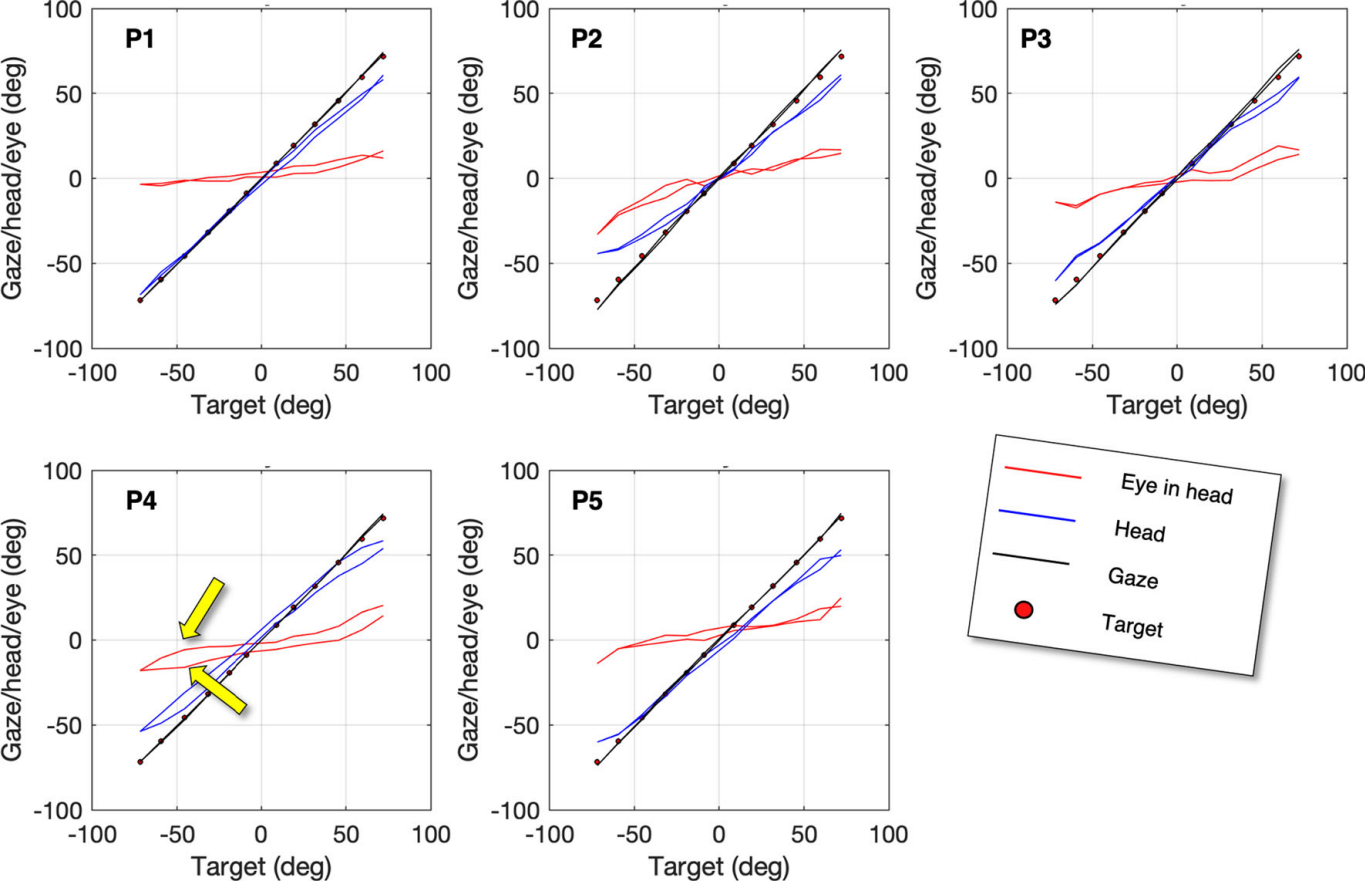
Gaze, head and eye orientation versus target direction. The five panels show eye-in-head orientations (*red line*), head orientations (*blue line*) and gaze orientation (*black*) for five participants recorded during subsequent fixation of the targets (see Fig. 1). The participants were instructed to fixate the targets from right to left and back (23 fixations in total, all targets except the most leftward target were fixated twice). The *two yellow arrows* indicate that P4 clearly uses different combinations of *eye-in-head* and *head-in-space* orientations during the two fixations of the targets

## Results

### Data quality

#### Accuracy

Figure 2 illustrates the eye, head, and gaze directions observed during 23 target fixations. The participants completed 12 fixations, starting from the rightmost target and progressing to the leftmost target, followed by 11 fixations to return to the rightmost target. Each of the five panels corresponds to eye and head orientation data from one participant. Notably, the gaze signal accurately represents the participants’ movements, as evidenced by the close alignment between the black line representing the gaze signal and the circles indicating the target locations.

The execution of the gaze task can be approached in various ways. For instance, in the case of P1, it is apparent that when fixating on the left targets, P1 fully turns his head towards those targets, while maintaining nearly 0^∘^ eye-in-head orientation. However, for the rightward targets, the head is not fully turned, and fixation is accomplished by rotating the eyes in the rightward direction. On the other hand, as shown by the yellow arrows in Fig. 2, P4 exhibits different combinations of head and eye orientations for the first and second fixation of a target.

According to Dunn et al. (2023), data quality of the eye-tracking signal should be reported. The gaze signal demonstrates excellent accuracy. Across participants, the mean absolute accuracy is 1.1^∘^ (range 0.6^∘^ to 1.9^∘^). These values are remarkably good considering the wide range of gaze angles involved in the study. It is important to note that during the calibration trial, the majority of the eccentric fixation positions were achieved through head rotation rather than eye rotation.

#### Precision

To assess the precision of the gaze signal, we employed a windowed method that accounts for blinks and saccades, eliminating the need for fixation classification (Hooge et al., 2018, 2022; Hessels et al., 2020). The root mean square (RMS) sample-to-sample deviation was computed per window, with each window consisting of 41 samples (equivalent to 205 ms). The median deviation across all windows was then determined. Across participants, the mean precision of the eye-in-head signal was 0.053^∘^ (range 0.036^∘^ to 0.077^∘^), the mean precision of the head signal was 0.021^∘^ (range 0.017^∘^ to 0.027^∘^) and the mean precision of the gaze signal was 0.051^∘^ (range 0.039^∘^ to 0.066^∘^).

#### Data loss

The Pupil Invisible has a variable sample frequency and does not report invalid samples in the data file. Conventional computation of data loss is problematic here. According to Hooge et al. (2022) one can also report the effective frequency instead of data loss. The effective frequency is operationalized as the number of valid samples divided by the time interval. The mean effective frequency (prior to resampling) of the eye-tracking signal is 200.54 Hz. We investigated the duration of the sample interval and it turned out that distribution was bimodal. The majority of the sample intervals are around 4 ms (sd = 0.08 ms) and 8 ms (sd = 0.08 ms). The mean effective frequency of the head signal is 250 Hz. After resampling, the mean effective frequency of the eye-in-head, head and gaze signals is 200 Hz.

### Replication of old studies

One of the key objectives of this paper is to investigate whether the general findings from the large eye–head gaze shift literature can be replicated using our setup. We first show that our eye-in-head and head signals look similar to the signals from the classic setups. We then investigate the relative timing between saccades and head shifts. Finally, we show the relation between amplitudes, velocities, and durations of gaze shifts and saccades.

**Fig. 3:**
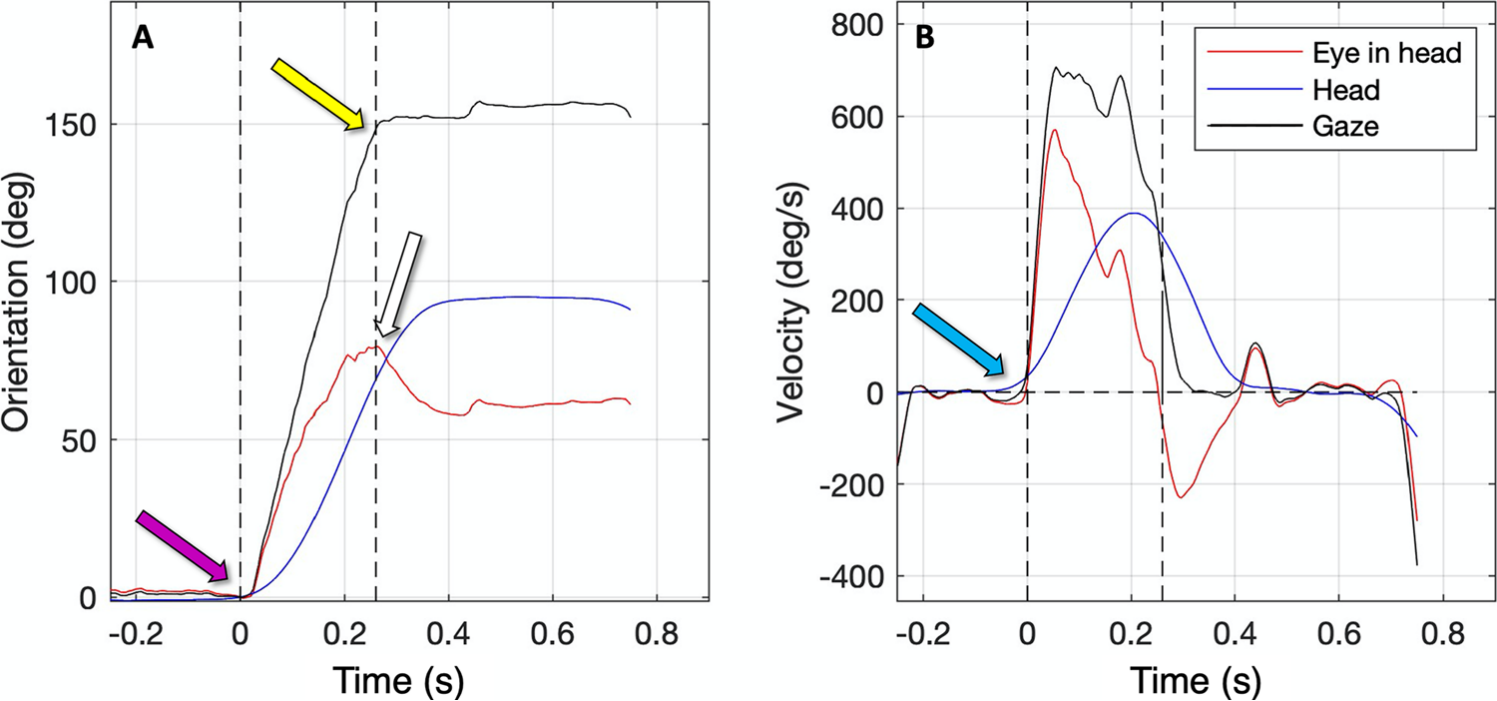
Eye-in-head, head and gaze signals. **A** Eye-in-head (*red*), head (*blue*), and gaze (*black*) versus time. The *purple arrow* denotes saccade start. The *white arrow* denotes saccade end and the start of the VOR phase. The *yellow arrow* denotes the end of the head-eye gaze saccade. The 150^∘^ gaze shift has about the same duration as the 80^∘^ saccade (±250 ms). The 90^∘^ head movement is much slower and has a longer duration of about 400 ms. **B** Eye-in-head velocity, head velocity, and gaze velocity versus time. The *blue arrow* clearly shows that in this specific example the head movement starts before the eye movement. Note that just before the saccade start the VOR is active. However, this is hard to see in this example. A better example is shown in Fig. 4B

**Fig. 4:**
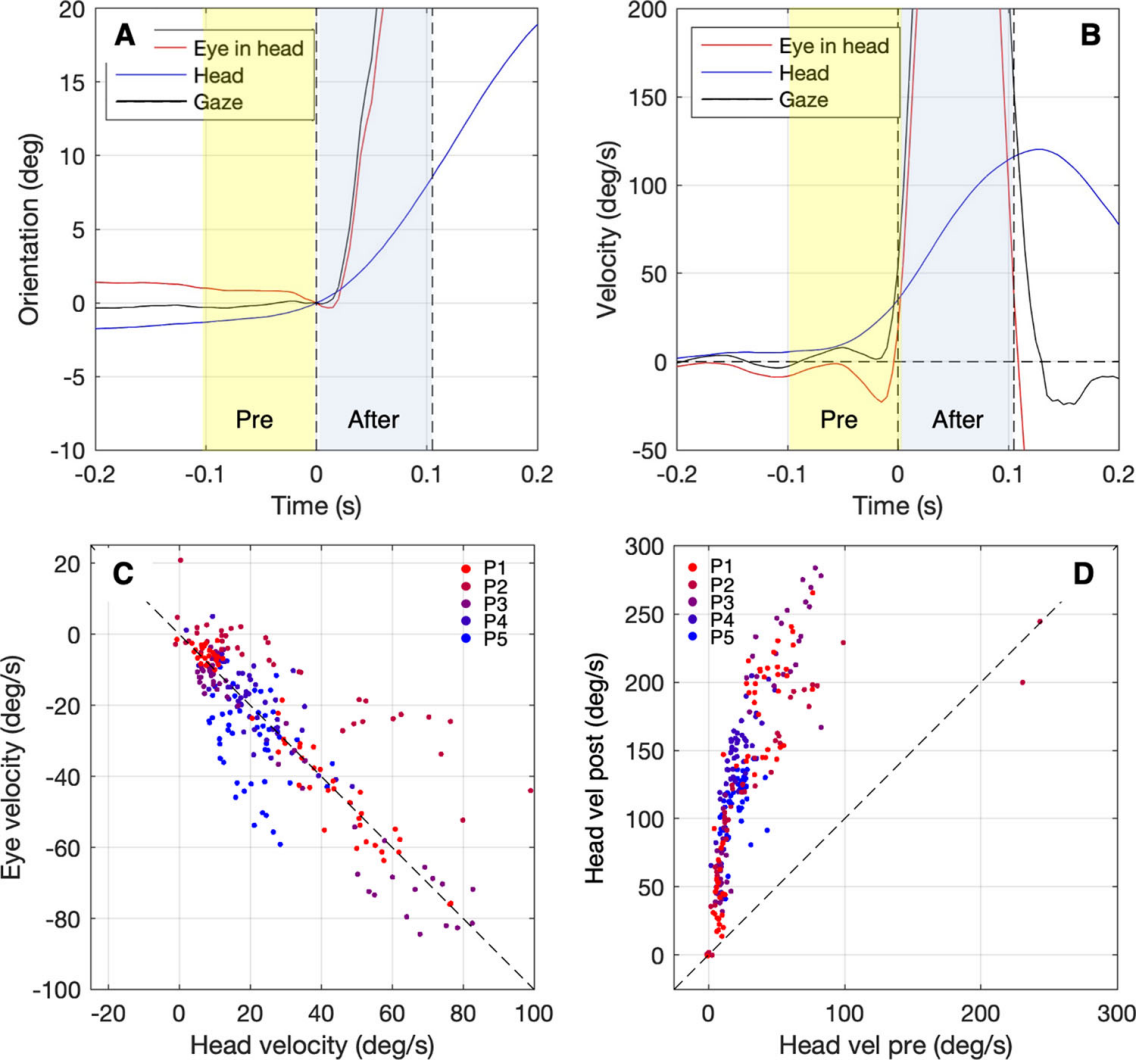
Relative timing of saccade and head movement. **A** Eye-in-head (*red*), head (*blue*), and gaze orientation (*black*) versus time for one movement. For this gaze movement, the head started moving before the saccade start. **B** Eye-in-head velocity (*red*), head velocity (*blue*), and gaze velocity (*black*) versus time for the same movement. *Light yellow* denotes the period from -100 ms until the saccade start; *light blue* denotes the period from the saccade start to +100 ms. **C** Mean eye velocity versus head velocity preceding the saccade for about 50 movements from five participants. Each *point* represents data from one gaze movement; *different colors* depict movements for different participants. Eye and head velocities were generally opposite, as illustrated by the points following the -45^∘^ line. This means that the VOR is active before the saccade starts. **D** Mean head velocity after the saccade start versus mean head velocity preceding the saccade start (for about 50 movements from five participants). Most of the points are far above the 45^∘^ line. This means that the head velocity after the saccade start is much higher and in the same direction as the head velocity before the saccade start. This is a clear indication that in most cases head movements already started before the saccade start

**Fig. 5:**
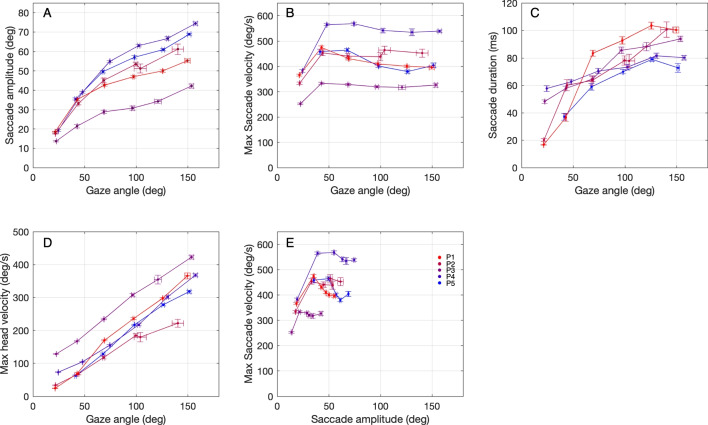
Durations, velocities, and amplitudes. **A** Mean saccade amplitude versus mean gaze angle. **B** Mean maximum saccade velocity versus mean gaze angle. **C** Mean saccade duration versus mean gaze angle. **D** Mean maximum head velocity versus mean gaze angle. The mean slope is 2.31 $s^{-1}$ ± 0.13 $s^{-1}$. **E** Mean maximum saccade velocity versus mean saccade amplitude. *Horizontal* and *vertical error bars* denote standard error of the mean. *Different colors* denote data from different participants

#### The relation between eye-in-head, head, and gaze signals

Figure 3 illustrates the eye-in-head, head, and gaze signals during a combined eye–head gaze shift. In this particular example, the head movement was initiated prior to the eye movement, indicated by the blue arrow in panel B. The vestibulo-ocular reflex (VOR) is active, clearly indicated by the opposite velocities of the saccade and head movement. During the saccade, the VOR is deactivated (Guitton & Volle, 1987), and both the eye and head rotate in the same direction. This is evident in panel B, where positive velocities are observed for both eye and head movements. After approximately 250 ms, the saccade comes to an end, and the VOR becomes active again (panel B again shows opposite velocities from 0.25 to 0.4 s). As a result, the gaze position stabilizes, maintaining a constant position.

The interaction between head movement and the eye movements (saccade and subsequent VOR) produces a rapid gaze shift (150^∘^) comparable in duration to the saccade (80^∘^). The gaze shift duration (250 ms) is much shorter than the head movement itself (which lasts over 400 ms). It is worth noting that rapidly rotating the head requires substantial energy and torque, considering that the average human head weighs between 4.3 to 5.3 kg (Clauser et al., 1969). The saccade, followed by a slower rotation back (due to the VOR), can be considered a mechanism that accelerates gaze shifts efficiently (Zangemeister & Stark, 1982).

#### The relative timing between saccades and head movements

From the literature, it is known that when the location and/or timing of the next visual target are unpredictable, the saccade starts 25–40 ms before the head movement (Zangemeister & Stark, 1982). In the case of predictable target locations, the head movement begins before the saccade (Moschner & Zangemeister, 1993). In the current experiment, the target locations are fixed and fully predictable, we expect that the head movement will start earlier than the saccade. Is this the case?

To investigate the relative timing between saccades and head movements, we cannot simply use the onset of the head movement and compare this time point with the saccade onset time. The reason is that some participants never fixate their head in this task, but continuously move their head. To investigate whether the head starts moving prior the saccade, we compare head and eye-in-head velocities prior to and after the saccade start.

Let’s consider an example to illustrate this phenomenon. If the head starts prior to the saccade we expect the following pattern. Prior to the saccade, the head already moves and the vestibulo-ocular reflex (VOR) is active, causing the eye and head velocities to be opposite in direction (Fig. 4B, yellow episode). Consequently, the gaze direction remains constant (Fig. 4A, yellow episode). After the saccade start, the VOR is deactivated, and the saccade and head velocities align in the same direction (Fig. 4B, blue episode). The head velocity prior to and after the saccade start have the same direction. The head velocity after the saccade start is much higher than prior to the saccade.

To analyze this for all gaze shifts, we calculated the mean eye and head velocities during the interval from 250 ms before until 750 ms after the saccade onset. Figure 4C illustrates the relationship between the mean eye-in-head velocity and the mean head velocity during this interval. These velocities exhibit opposite directions and roughly follow a unity line with a negative slope. This indicates that the head already moves and the VOR is active. Additionally, Fig. 4D presents the mean head velocity after the saccade start versus the mean head velocity estimated prior the saccade start. As expected, the head velocity is much higher after than prior to the saccade start. This analysis reveals that in the conducted experiment, the head movement tends to be initiated prior to the saccade for the vast majority of the observed gaze movements.

#### Gaze, head and saccade amplitude velocity, and duration

In Fig. 5A, we demonstrate that the saccade amplitude exhibits a less than proportional increase with gaze angle (as in Fig. 5A, Freedman 2008). This finding indicates that the saccade’s relative contribution to the overall gaze movement diminishes as the gaze angle increases. In Fig. 5B, it can be observed that the saccadic peak velocity increases if the gaze angle increases from 25^∘^ to 50^∘^. For larger gaze angles, velocity slightly decreases and then remains constant for larger gaze angles. Figure 5E shows a similar relation for saccade amplitude. Figure 5B and E remarkably resemble the patterns shown in Fig. 7 in Freedman (2008). Furthermore, Fig. 5C illustrates that saccade duration increases with gaze angle but eventually levels off. The prolonged saccade duration is attributed to saccades traversing longer distances without higher maximal velocities (Fig. 5B and E). The levelling off of saccade duration for very large gaze angles is due to the diminishing increase in saccade size at those angles. It should be noted that the physical size of the saccades is limited to approximately 80^∘^ (Collewijn et al., 1988). Figure 5D exhibits the proportional relationship between maximum head velocity and gaze angle (with an average slope of 2.31$s^{-1}$ ± 0.13$s^{-1}$). Our findings are consistent with those of prior studies such as Gresty (1973); Guitton and Volle (1987); Land (2004).

## Discussion

### Summary of results

In this study, our aim was to replicate common findings from the large eye–head gaze shift literature using a novel setup consisting of a wearable eye tracker, a high-speed camera, and fiducial marker technique. We successfully replicated a number of main findings. We observed the following:

1. In the case of predictable target locations, the movement of the head starts before the saccade.
2. We noticed a decrease in the relative contribution of the saccade for larger gaze angles.
3. For substantial gaze movements, the maximum speed of saccades remains constant.
4. The maximum speed of head movements increases proportionally with the gaze angle.

Furthermore, we conducted an analysis of the data quality pertaining to the gaze signal. The accuracy of the gaze signal was found to be good, the mean absolute accuracy was 1.1^∘^ observed within a gaze range of up to 140^∘^. Precision, quantified by the RMS-S2S deviation, exhibited a mean value of 0.051^∘^. We were not able to estimate data loss as the Pupil Invisible generates eye-tracking data even when participants close their eyes. The effective frequency of the eye-in-head signal was 200.54 Hz prior to resampling.

### Requirements for an eye tracker and a head tracker

What are the essential requirements for a setup to accurately capture and record large gaze movements? We will evaluate the following aspects: precision, sampling frequency, and the ability to detect blinks.

#### Precision

Despite technological advancements, the current bottleneck in modern combined eye- and head-tracking setups lies within the wearable eye tracker. In contrast, the previous generation of setups that utilized coils to measure eye and head orientation did not face this limitation. The coil signal provided exceptional precision, allowing for easy analysis of the dynamics of eye and head movements. The modern setup suffers from two primary issues in comparison to the previous. First, the precision of the wearable eye-tracking signal is significantly lower than that of coils. Second, when the focus is on studying gaze movement, coil setups have an advantage. The coil measures gaze directly in space, whereas the combination of a wearable eye tracker and a head tracker determines gaze indirectly by combining the eye-in-head signal with the head-in-space signal. In the latter case, the imprecision of the gaze signal is determined by the imprecision of both the eye and the head tracker. Whether the imprecision of the new setup with a wearable eye tracker is problematic depends on the use case.

#### Sampling frequency and bandwidth

The bandwidth of saccades is estimated to be around 75 Hz (Bahill et al., 1981, 1982). To accurately capture the dynamic properties of saccades, it is recommended that the recording frequency be set at a minimum of 150 Hz, which is twice the Nyquist frequency. However, recording at even higher frequencies is preferable. Higher recording frequencies, exceeding 150 Hz, offer additional benefits such as more precise determination of saccade start and endpoint. Preferably, the sampling frequency for the head tracker should be similar to that of the eye tracker because both the head-in-world signal and eye-in-head signals are used to compute the gaze signal. If the head tracker has a lower sampling frequency than the eye tracker, one may choose to upsample the frequency. Because the head has a large mass, the head-in-world signal is much smoother (less jerky) than the eye-in-head signal. In general, if the head movement is caused by the participant, upsampling the head signal is not problematic. Upsampling may be problematic if the head movement is also caused by external (high frequency) perturbations (e.g., Boulanger et al. 2012). In that specific case, the sampling frequency should be high enough to be able to pick up all frequencies present in the head signal.

When the dynamics or precise determination of saccade start and endpoint are not of interest, an eye tracker with a lower sampling frequency can be used. There are multiple previous examples of such research: Land (2004) investigated gaze shifts including trunk rotations. He recorded eye-in-head and the scene with a self-built wearable eye tracker during food preparation and driving. Eye and scene camera recorded at 50 Hz. His method allowed him to study only the larger gaze shifts because they have long duration. Kothari et al. (2020) used a pair of Pupil Labs eye-tracking glasses (120 Hz), an inertial measurement unit (IMU), and a 3D stereo camera to produce a data set of hand coded events (e.g., gaze fixation, gaze shift, blink and gaze pursuit). A final example is by Fang et al. (2015), who used a 60-Hz wearable eye tracker (NAC EMR-9) and 60-Hz head tracker (Polhemus Fastrak) to investigate head and eye-in-head orientations during visual search in a large visual stimulus. The focus of Fang’s study was on the spatial (not the temporal) aspects of eye, head, and gaze movements.

#### Blinks

The version of the Pupil Invisible that we have used has a drawback for researching large gaze shifts. The Pupil Invisible always reports a gaze direction, even if the eyes are closed or when the participant blinks. However, during natural behavior, there are often blinks that occur during significant gaze movements (Evinger et al., 1994). Therefore, we instructed the participants not to blink during these substantial gaze movements and advised them to blink freely during gaze fixations if needed. The newer version of Pupil Cloud (v5.7) includes a signal that aids in identifying blinks.

### Head trackers compared

In this article, several methods for capturing head orientation and/or position have been mentioned (e.g., potentiometer, magnetic tracker, infrared markers, ultrasonic sound source localization, cyclorotational coils, inertial measurement unit (IMU), and fiducial marker technology (ArUco)). Each of these methods has its advantages and disadvantages. The potentiometer is limited to rotation around a single axis, while the other mentioned techniques are not. Some of the techniques are easy to implement if certain facilities are already available in a lab. This applies, for example, to cyclorotational coils. If there is a three-field setup with lock-in amplifiers with enough inputs, a cyclorotational coil can be attached to the head (Epelboim et al., 1995) or a biteboard (Steinman, 1996) to determine the 3D orientation (not the position) of the head. IR marker systems (e.g., Optotrak, Optitrack) and magnetic trackers (e.g., Flock of Birds, Polhemus) are well suited for measuring 3D position and 3D orientation. Unlike all the other mentioned techniques, the current fiducial marker technology does not work in the dark. Another disadvantage of fiducial marker technology is that if markers are placed in the world, they can visually interfere with any visual task. However, the latter does not apply to the setup in this article. Recently, Ayala et al. (2023) developed the invisible ArUco marker that is visible under IR-light. Some techniques are inexpensive (IMUs and fiducial marker technology), while others can be quite costly (Optotrak). In what follows, we discuss how the two inexpensive techniques can be employed and whether one of the two is superior.

The ArUco marker technology can be very precise and accurate. The limitation of the current setup (marker on the head, camera attached to the ceiling) is that the main movement has to occur within a specific volume. The size of the ArUco marker (bigger is better), as well as the temporal and spatial resolution of the camera (spatial resolution recording frequency trade-off), determine the sampling frequency and resolution of the measurement. A high-quality camera and a better lens can make the setup more expensive. As applied in this article, this technique is very suitable for determining the head position and orientation of a participant sitting on a chair. We used one marker, but the number of markers is not limited to one. A useful multi-marker application could be measuring the head orientation of three participants concurrently while sitting at a round table having a conversation. It could also be a useful tool for measuring head orientation and position in an interaction experiment as in Hessels et al. (2023). Due to the fixed camera, the ArUco marker technology is not suitable when participants are freely moving or outside the lab. A good reason to use the ArUco marker technique is that it is widely used, free, and there is a lot of information about how to use it available on the Internet. While the ArUco technique is not perfect, many researchers work on resolving the existing issues with the technique. For example, in theory, the 3D orientation and 3D position of a marker can be uniquely determined based on four non-collinear but coplanar points. In practice, the situation is less clear in non-ideal conditions, such as when the depicted marker is small or when the marker is at a distance significantly greater than the focal length of the camera lens. This problem has been addressed by Ch’ng et al. (2020). In addition, while the library indeed reports 6 degrees of freedom from a single marker, it is known that there is significant noise if markers are small (see, e.g., Poroykov et al. , 2020; Kalaitzakis et al. 2021). Regarding the size of the ArUco marker in the present study, Poroykov et al. (2020) showed (in their Fig. 3) that if the marker area exceeds approximately 1500 pixels, the error in distance estimation is less than 3 mm. In our study, the marker area exceeded 80,000 pixels.

The advantage of IMU technology is that it is not dependent on external devices such as cameras attached to the ceiling. That means that IMU technology can also be used with free-moving participants or outside the lab. With an IMU, both position and orientation can be estimated. However, this estimation is indirect; the IMU uses accelerometers for position and gyroscopes for head orientation. Kothari et al. (2020) used an inexpensive IMU (MPU-6050) with a three-axis accelerometer and a three-axis gyroscope. This IMU estimates the orientation relative to the initial position at the start of the measurement. It is known that orientation estimations are prone to drift. Through various methods including calibration and fine-tuning during post-processing, the error was reduced to 7^∘^ ($\sigma$ = 8.34^∘^) for short recordings. For longer recordings, the error tends to increase. Frequent head rotations can also lead to an increase in head orientation error. To limit the error, according to Kothari et al. (2020), the IMU should be reset after a few head rotations. One reason is that accelerometers seem not very suitable for estimating orientation during dynamic tasks because they cannot distinguish between gravitational acceleration and acceleration of the moving head.

In a recent literature overview, Nazarahari and Rouhani (2021) reviewed many studies on sensor fusion in magnetic and inertial measurement units (MIMU). A MIMU is an IMU with an additional geomagnetic field sensor that can help mitigate drift. They describe in their article that MIMUs do not provide accurate pose estimation for three reasons. The first reason is that gyroscopes are not suitable for orientation estimation during prolonged tasks. The second reason is that magnetometer-based estimations are sensitive to metal in the surroundings, and the third reason is the previously mentioned issue that accelerometers do not work well during dynamic tasks. The suggested sensor fusion techniques are meant to produce a MIMU that delivers the orientation accurately. An interesting point in the review is that Nazarahari and Rouhani (2021) advice to use a benchmark for MIMU performance that at least contains the following: “The true orientation is recorded synchronously with MIMUs using a reference system such as the camera motion-capture system”. We think that a camera and one ArUco marker can do this.

### Frames of reference and terminology

In the eye movement literature, the terms *eye movement*, *saccade* and *gaze movement* are often used sloppily or interchangeably (Hessels et al., 2018). For example, it often occurs that the *saccade* is referred to as an eye movement. We believe this occurs because in specific research contexts (e.g., reading, attention or working memory), researchers primarily use head-fixed eye-tracking setups with static stimuli, where the saccade is the only eye movement. However, it is important to note that the saccade is not the only type of eye movement observed when using dynamic instead of static stimuli or employing remote or head-free eye-tracking setups instead of a head-fixed eye-tracking setup. Then a wide range of eye movements may occur, including smooth pursuit, vergence, and OKN. VOR may occur if the observer moves the head, or the head is moved by someone or something else. It is crucial to be specific and refer to the saccade as a distinct eye movement rather than simply labeling it as an eye movement. We strongly advocate for this level of precision in terminology (Dunn et al., 2023). Note that all eye movements mentioned above are usually defined in a head-fixed reference frame.

A more challenging problem is the use of the terms *gaze point* and *gaze direction* (Hessels et al., 2018). When a remote eye tracker is used, there is some freedom to move the head. In principle, the movement recorded by the remote eye tracker is a gaze movement because it records the point of regard in a world-fixed reference frame. When the participant fixates a point on the screen and simultaneously moves (translates and/or rotates) the head, the eye tracker should not detect a gaze movement. However, the participant does make an eye rotation relative to the head to fixate a point on the screen. In the context of the remote eye tracker (which might be more appropriately termed a gaze tracker), authors often use the head-fixed eye movements terminology instead of the world-fixed gaze terminology. They continue to refer to saccades, smooth pursuit, and fixations. Often this is not a problem because the reader understands what is being discussed, but it can also become confusing. Discussions may then arise about whether a smooth pursuit episode can also be a fixation or not (for an extensive discussion see, Hessels et al. 2018).

Today, an increasing number of wearable eye trackers are being used in research. Describing signals from wearable eye trackers can become complicated when there is no awareness of the reference frames. Many users of wearable eye trackers use so-called gaze overlay videos to observe their participants’ behavior. A gaze overlay video shows a marker representing the point of regard in a head-centered frame of reference, because the scene camera is attached to the head. The absolute movements of the gaze marker in the movie frame are eye movements relative to the head. Because the viewer of the gaze overlay film, which might be better called an eye movement overlay film, can experience with their own visual system that a participant fixates on an object in the world moving relative to the head, they interpret the displayed behavior in terms of gaze in the world. Through proper reporting in eye-tracking studies, the reference frame of the signal should be clear to the reader (Dunn et al., 2023).

In the present article, the world is the reference frame for both head and gaze movements, and for eye movements, the reference frame is the head. We are not proponents of imposing mandatory terminology, but at least defining the terms used correctly once in the beginning of an article seems like a good practice to us. Many new users of wearable eye trackers often carry a history of head-fixed and remote eye tracking with them and they keep using the old terminology.

A quick review of literature reveals that many authors are aware of the frames of reference, but use a variety of terms interchangeably. For example, Kothari et al. (2020) uses the terms gaze pursuit and gaze fixation, Land (2004) uses gaze saccades and gaze rotation. Additionally, Freedman (2008) consistently uses gaze shift, Fuller (1992) employs gaze fixation, gaze saccade, and gaze shift, while Goossens and van Opstal (1997) use gaze fixation, gaze saccade, gaze shift, and gaze movement. Evinger et al. (1994) uses both saccadic gaze shifts and gaze shift. We suggest following the terminologies used by e.g. Land (2004); Goossens and van Opstal (1997) and Kothari et al. (2020). Land explains that head saccade refers to the rapid movement of the head accompanying an eye saccade, while gaze saccade denotes the change in gaze direction resulting from a combined eye and head saccade. Gaze pursuit and gaze fixation should be incorporated into the terminology. What we propose here is not new for researchers from the eye–head gaze shift field. It is new for researchers coming from head-fixed and from remote eye tracking. The old eye-related terms (e.g., saccade, pursuit) refer to the eye-in-head frame, gaze terminology refers to the reference frame of the world and head-related terms are still ambiguous. Are they in a frame of reference of the body or the world? This can be solved easily, researchers can always define their terminology.

## Conclusions

Building a novel setup with a wearable eye tracker and fiducial marker technology, we replicated many findings from the large eye–head gaze shift literature. We conclude that our new inexpensive setup is good enough to investigate the dynamics of large gaze shifts. Besides research on the control of saccades and head movements during gaze shifts, this setup could also be interesting for extending existing research on human interaction (e.g., Hessels et al. 2023).

## Open practices statement

The eye-tracking, head and gaze data and a routine to convert Pupil Invisible eye-tracking data from pixels to degrees are available here: https://github.com/dcnieho/HoogeetalEyeHeadGazeShifts. The experiment was not preregistered.

## Footnotes

1 The measure underlying this number is still debated. For an interesting history of peripheral vision limits, see Strasburger (2020). For a review on more recent measures of the FOV, see Simpson (2017)
